# Malaysian Journal of Medical Sciences’ Performance Status in 2017 and the Challenges

**DOI:** 10.21315/mjms2018.25.6.1

**Published:** 2018-12-28

**Authors:** Nour Azimah Zulkapli, Jafri Malin Abdullah

**Affiliations:** 1 Production Editor, Malaysian Journal of Medical Sciences, Universiti Sains Malaysia, 11800 USM, Pulau Pinang, Malaysia; 2 Chief Editor, Malaysian Journal of Medical Sciences, Universiti Sains Malaysia Health Campus, 16150 Kubang Kerian, Kelantan, Malaysia

**Keywords:** challenge, performance, submissions, workload, administrator

## Abstract

This report presents a review of the Malaysian Journal of Medical Sciences’ (MJMS) performance status throughout 2017, which covers the submission pattern of original manuscripts by month, the geographical distribution of submitting authors, the types of manuscripts and overall acceptance/rejection rates.

As the years progress, MJMS continues to receive an escalating number of manuscript submissions. This contributes to an ever-increasing workload, which makes administrative tasks continually more challenging. Although the manuscript submission platform seeks to minimise the pre-publication workload of the journal administrator, it is still a time-consuming task, particularly when authors seek exclusive attention for their submitted manuscripts.

## Introduction

As expected, the number of original manuscripts sent to the Malaysian Journal of Medical Sciences (MJMS) via our electronic submission system, ScholarOne Manuscripts™, has shown an increase of 17.5%, from 301 manuscripts in 2016 (1) to 365 manuscripts in 2017. This increment proves that the existence of MJMS is recognised by medical peer groups in other parts of the world. Moreover, all researchers are concerned with publication in scientific journals, as they are the most important medium for the dissemination of their work (2).

## Submission Pattern by Month

**Figure 1:**
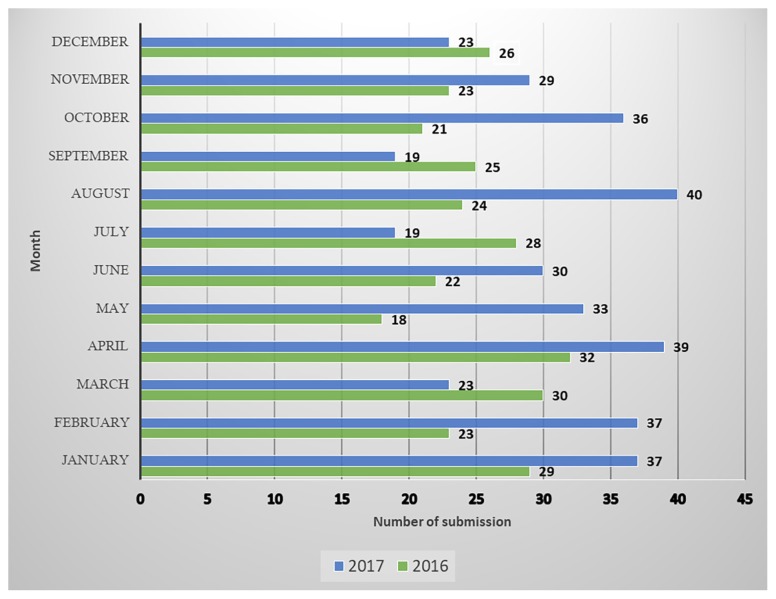
Information based on original manuscripts submitted via ScholarOne Manuscripts™ system in 2016 and 2017 *Source*: https://mc.manuscriptcentral.com/maljms

2017 witnessed an unpredictable number of monthly manuscript submissions. On average, at least 30 manuscripts were received each month, with a high of 40 received in the month of August. Other peak months included January, February, April and October (Figure 1).

## Submission by Geography

Malaysia has remained the top manuscript contributor, with 154 submissions (42.2%). Table 1 shows that the number of manuscripts submitted by Malaysian authors is more than double those submitted from Iran. As seen in Table 1, India has fallen to third place, compared to 2016, when the country used to be listed in second place, followed by Iran in third (1). Indonesia is in fourth place with 32 submissions, followed by Nigeria with 22. The rest included those with a minimum of one submission.

## Submission by Manuscript Type

**Figure 2:**
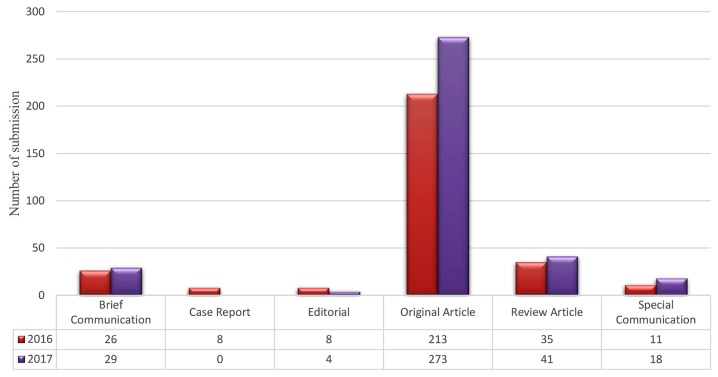
Number of manuscript submissions by type Source: https://mc.manuscriptcentral.com/maljms

Out of 365 manuscripts submitted in 2017, ‘Original Article’ manuscripts were the most common, with 273 submissions in 2017 compared to 213 submissions in 2016 (1). ‘Review Article’ manuscripts were the second most submitted, followed by ‘Brief Communications’ manuscripts (Figure 2).

## The Acceptance/Rejection Rate of Manuscripts

**Figure 3:**
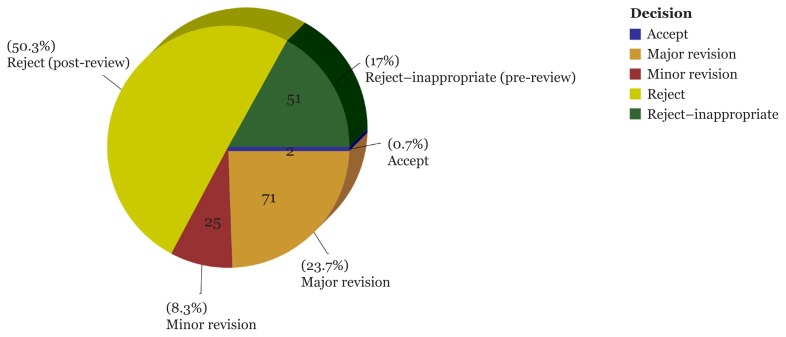
Percentage and number of manuscripts by decision submitted between Jan 1, 2017 and Dec 31, 2017; decision date is between Jan 1, 2017 and Dec 31, 2017 Grouped by: Manuscript Decision *Source*: https://mc.manuscriptcentral.com/maljms

Figure 3 was constructed based on the data of manuscripts with decision dates between Jan 1, 2017 and Dec 31, 2017. The percentage of rejections occupies a huge portion in the pie chart where 50.3% of rejections were due to decisions made by the Editor-in-Chief based on collective reviews, while 17% were due to the inappropriateness of the scope or content of the study. Whereas, 23.7% were sent back for major revision, which is proportionately high compared to 8.3% slated for minor revision. Only 0.7% of post-reviewed manuscripts were accepted for publication. These figures show that MJMS is really concerned with quality rather than quantity.

## Challenges Faced by the Journal Administrator

Submission of research manuscripts is a much easier process today because of online submission systems like ScholarOne, Editorial Manager and Evise^®^ used by publishers all over the world (3). These systems work in a largely similar way, and are meant to make the publishing process more efficient and readily accessible to both authors and reviewers.

On the other side of things, however, the journal administrator faces increasing challenges along the journey of journal publication, when finding solutions and technical troubleshooting become additional tasks alongside juggling the responsibilities of being a copy editor, production editor and system administrator.

One recurring difficulty is time pressure. This is defined as the subjective feeling of having less time than required to complete a task. It arises each time the journal administrator has to perform a long list of tasks which must keep moving in order to meet deadlines. This includes having to respond to queries by authors through emails while the queue of new submitted manuscripts continues to build up, and keeping authors updated when they insist on exclusive attention. The reality is that everyone wants their manuscript to be entertained first. Last but not least, the administrator must deal with imperfect manuscripts which do not meet the journal’s requirements as given in its guidelines.

Based on a study by Moore and Tenney (4), time pressure generally impairs performance because it places constraints on our capacity for thought and action which limit exploration and increase reliance on well-learned or heuristic strategies. Thus, time pressure increases performance speed, but at the expense of quality.

## Figures and Tables

**Table 1:** Number and percentage of manuscripts submitted, listed by country of submitting author

Country of Submitting Author	Manuscripts	Percentage
Malaysia	154	42.2%
Iran (the Islamic Republic of)	60	16.4%
India	47	12.9%
Indonesia	32	8.8%
Nigeria	22	6.0%
Iraq	5	1.4%
Saudi Arabia	5	1.4%
Turkey	5	1.4%
Pakistan	4	1.1%
Brunei Darussalam	3	0.8%
Thailand	3	0.8%
Egypt	2	0.5%
Germany	2	0.5%
Italy	2	0.5%
Japan	2	0.5%
Nepal	2	0.5%
Oman	2	0.5%
Algeria	1	0.3%
Aruba	1	0.3%
Australia	1	0.3%
Bangladesh	1	0.3%
Cyprus	1	0.3%
Kazakhstan	1	0.3%
Mexico	1	0.3%
Palestine, State of	1	0.3%
Russian Federation	1	0.3%
South Africa	1	0.3%
Spain	1	0.3%
United Kingdom of Great Britain and Northern Ireland	1	0.3%
Total	365	100%

Source: https://mc.manuscriptcentral.com/maljms
